# Lymphocytic Esophagitis: Diagnosis and Management in the Emergency Department vs Initial Suspicion of Eosinophilic Esophagitis

**DOI:** 10.7759/cureus.30300

**Published:** 2022-10-14

**Authors:** Wesley D Banks, Christian Cassar, Philip Cassar

**Affiliations:** 1 Emergency Department, St. Joseph Medical Center, Bethpage, USA; 2 Gastroenterology, SUNY Downstate Medical School, Brooklyn, USA; 3 Gastroenterology, St. Francis Hospital, Roslyn, USA

**Keywords:** endoscopy, auto-immune, esophagus, emergency gastroenterology and endoscopy, lymphocytic esophagitis

## Abstract

Lymphocytic esophagitis is an increasingly prevalent yet poorly understood condition that is highly disruptive to daily living. The presentation often includes dysphagia, but dysarthria and narrowing of the esophageal lumen may be seen as well. In this case, a 66-year-old female presented to the Emergency Department complaining of dysphagia for several weeks in addition to associated discomfort with the loss of ability to swallow solid foods.

## Introduction

When dealing with a seemingly benign presentation in the Emergency Department (ED), it is important to resist the temptation to jump to conclusions and diagnose the “most common cause.” The presentation of dysphagia may be due to a wide range of conditions, including but not limited to paralysis of the swallowing muscles, esophageal web, eosinophilic esophagitis (EoE), peptic stricture, esophageal malignancy, and many types of tumors [1]. The rare presentations, however, can be most difficult to diagnose and treat due to the sneaking masquerade of more benign and seemingly common causes. Lymphocytic esophagitis (LyE) is a rare condition characterized histologically by high numbers of esophageal intraepithelial lymphocytes without significant granulocyte infiltration, in addition to intercellular edema [2]. This condition causes a significant narrowing of the upper, middle, and lower esophageal lumen and predominantly creates consistent difficulty in swallowing. The specific physiological changes caused by the disorder will be discussed in detail later. LyE is definitely a rare condition, but one that can have significant consequences if gone undiagnosed or even confused with a close counterpart such as EoE. This case study will contain some discussion of the difference between LyE and EoE, as it is important to note the distinction between the two.

## Case presentation

**Figure 1:**
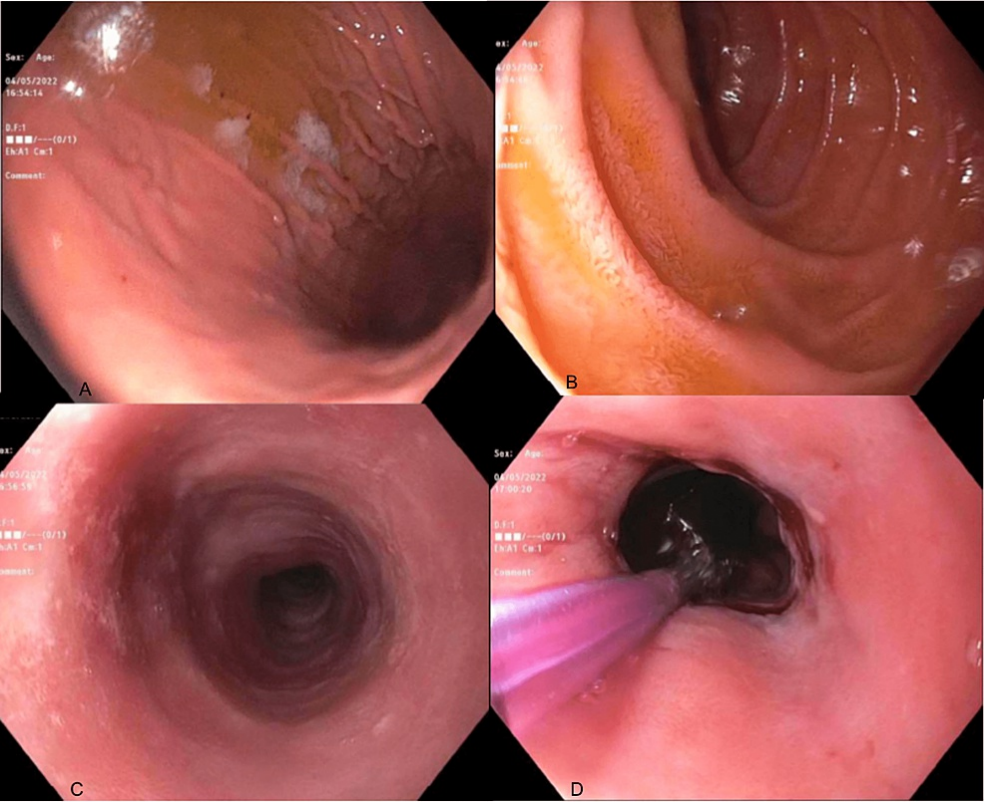
Patient Endoscopy A) shows circumferential folds of the stomach, B) shows the duodenum, C) shows edema and white spotting of the esophagus, and D) shows narrowing of the esophagus that is being prepared for balloon dilation. These are some of the hallmark findings on endoscopy of lymphocytic esophagitis.

In this case, a 66-year-old female presents to the primary care physician (PCP) with a complaint of dysphagia for six weeks. The patient reports to the PCP that she is unable to successfully eat solid foods, and has been on a strict liquid diet for the duration of the presentation. She was scheduled for a follow-up with gastroenterology in two months’ time and had recently been cleared by her otolaryngologist after laryngoscopy was unremarkable. However, the PCP decided to refer the patient to the ED due to her recent weight loss and significant discomfort for a more emergent intervention. The patient has a past medical history significant for esophageal web and received balloon dilation of 10 mm approximately 1 year prior to this episode which showed moderate mucosal disruption. An esophagram performed in the ED by gastroenterology (GI) showed a narrowing of the lumen in the proximal esophagus, and immediate endoscopy was recommended. On endoscopy, the patient had mucosal changes including a ringed esophagus, circumferential folds, congestion (edema), punctate white spots, and stenosis in the upper, middle, and lower thirds of the esophagus as well as the gastroesophageal junction. Figure 1 depicts the images from the endoscopy.

Following endoscopy by GI, an initial diagnosis of EoE was made due to the edematous presentation and esophageal web from the past history. During the procedure, however, random sample biopsies were taken and sent to pathology for analysis. Upon return, the pathologist determined that the diagnosis was LyE. The findings that confirm this diagnosis demonstrated increased intraepithelial, as well as submucosal, lymphocytes with relatively few neutrophils in the upper, middle, and lower esophagus. Neither eosinophils were found to be prominent, nor was there evidence of viral inclusions. The silver stain was negative for fungal organisms as well. The pathology report was also noteworthy for increased T cells with an admixture of CD4-positive and CD8-positive cells. These findings, markedly in the absence of eosinophils, point toward LyE as a definitive diagnosis which has a better response to treatment historically than does EoE [1]. In the case of this patient, symptomatic management was deemed to be the best approach. The patient was started on pantoprazole, a proton pump inhibitor (PPI), and prednisone, an oral corticosteroid, status-post balloon dilation and discharge from the hospital. Outpatient management and follow-up are important, and dysphagia is the leading presentation of concern as far as recognizing the recurrence of chronic LyE.

## Discussion

It is important to understand the difference between LyE and EoE when considering the differential diagnosis of esophageal pathology, specifically dysphagia, and luminal narrowing. LyE is a rare condition characterized histologically by high numbers of esophageal intraepithelial lymphocytes without significant granulocyte infiltration, in addition to intercellular edema [2]. EoE is a chronic, immune-mediated, or antigen-mediated esophageal disease characterized by symptoms related to esophageal dysfunction and eosinophil-predominant inflammation. The dominant antigens that mediate this disease appear to be food-based [3]. There is also a strong response to eosinophils in EoE. In other words, LyE is more regarded as an autoimmune response, whereas EoE is an allergic reaction most often in response to the food that can become chronic. Both diseases may have similar physical appearances, and both can cause chronic and lasting complications, but the approach to treating an allergic reaction vs an autoimmune disorder is vastly different. The prevalence of LyE is estimated as 0.1% in an adult population, 5.7% in a pediatric population, 8.6% among adult patients with food bolus impaction, and 0.2% among adult patients with dysphagia and/or food bolus impaction [4]. It is also noteworthy that women between the ages of 40 and 59 seem to be the highest at-risk population [5]. The treatment of LyE remains without solid guidelines and is mostly aimed at treating the patient symptomatically. According to more recent literature, gastroesophageal reflux disease (GERD) may be a large contributing factor to the pathology by means of acid reflux and motility issues [6]. In the patient discussed previously, an X-ray-modified barium swallow showed no evidence of penetration, aspiration, or stasis with thin fluids, puree, or even solids. It is this atypical presentation with a rather inconsistent course that presents such a challenge for the treatment of such a disorder. Since the treatment for LyE is mostly symptomatic, the most common, and seemingly empirical, treatment of the disease involves oral steroids, PPI, and balloon dilation. The mechanism of the medications is worth exploring. The PPI mechanism of action targets the gastric H+, K+-ATPase, aka proton pumps, that supplies acid for the stomach and irreversibly binds to the enzymes [7]. There are multiple pathways that initiate the stimulation and release of gastric acid in the stomach. Older classes of medications such as histamine receptor antagonists, a classic antacid medication target and prevents the stimulation of receptors that eventually lead to the activation of H, K-ATPase. This mechanism of action has poor long-term efficacy and has been shown to even be overridden by food consumption [8]. PPIs, however, essentially bypass the multiple mechanisms of stimulation and block the central pathway source of release. It is for this reason that PPIs have been shown to be most effective in both the short- and long-term management of patients being treated for stomach acid-induced pathology.

Oral steroids inhibit transcription factors that control the synthesis of pro-inflammatory mediators, including macrophages, eosinophils, lymphocytes, mast cells, and dendritic cells. By inhibiting genes responsible for the expression of cyclooxygenase-2, inducible nitric oxide synthase, and pro-inflammatory cytokines, including tumor necrosis factor-alpha and various interleukins [9]. By essentially suppressing the immune system, oral steroids are considered a staple treatment in autoimmune pathology such as LyE. Balloon dilation is another useful tool in the treatment of many esophageal narrowing disorders, including LyE. Balloon dilation is ultimately used for two reasons; the goal is to relieve a patient’s dysphagia and to prevent the recurrence of stricture. As esophageal stenosis worsens over time, the dysphagia progresses from solid to semi-solid to liquid foods. The etiology of the esophageal stricture usually can be identified using radiographic modalities and is confirmed by endoscopic visualization and tissue biopsy. The mechanics of esophageal luminal dilation results in circumferential stretching, or frank splitting, of the stricture. Balloon dilators deliver a force radially and uniformly across the entire length of the stricture, which significantly reduces shear stress [10].

## Conclusions

The purpose of this case study is to recognize the rarity of LyE and demonstrate the most effective way to diagnose, manage, and treat its presentation. The similarity to EoE on endoscopic exams makes the diagnosis challenging, and a good differential is required to at least consider LyE. Biopsy is ultimately the best way to conclusively diagnose the disorder, and treatment has been shown to have a better response for LyE than that of its commonly misconceived EoE counterpart. A course of PPIs and Oral Corticosteroids, Status-post balloon dilation, seem to be the most closely “empirical” treatment of LyE, but definitive guidelines are still needed for the proper management of the disorder beyond symptomatic management.
